# Effects of Running Fatigue on Lower Limb Joint Kinematics and Kinetics in Female Genu Valgum Individuals: A Comparative Study

**DOI:** 10.1155/abb/8842670

**Published:** 2025-10-07

**Authors:** Xiaoyu Jian, Dong Sun, Yufan Xu, Chengyuan Zhu, Xuanzhen Cen, Yang Song, Fengping Li, Gusztáv Fekete, Yaodong Gu

**Affiliations:** ^1^Faculty of Sports Science, Ningbo University, Ningbo, China; ^2^Department of Biomedical Engineering, Faculty of Engineering, The Hong Kong Polytechnic University, Hong Kong, China; ^3^Department of Materials Science and Machine Design, Audi Hungaria Faculty of Automotive Engineering, Széchenyi István University, Győr, Hungary

**Keywords:** genu valgum, joint angle, joint moment, lower limbs, running-induced fatigue

## Abstract

Individuals with nontraumatic genu valgum (GV) may be at an increased risk of anterior cruciate ligament (ACL) injuries. This study investigates the impact of fatigue on lower limb biomechanics in individuals with GV compared to healthy controls. A total of eight female participants with GV and eight female healthy controls were recruited. All participants completed a running-induced fatigue protocol. Kinematic and kinetic data were collected, followed by statistical analysis using independent and paired-samples *t*-tests to compare between-group and within-group differences, respectively. The results demonstrated that compared to the control group, the fatigued GV group exhibited significantly greater hip flexion angles and hip flexion moments, hip internal rotation angles and hip internal rotation moments, knee flexion angles, knee internal rotation angles, and knee external rotation moments. Similarly, individuals with GV exhibited increased ankle plantarflexion angles, ankle dorsiflexion moments, ankle external rotation angles, and ankle external rotation moments. Moreover, the GV group displayed greater knee adduction angles, hip abduction angles, and ankle adduction angles than their healthy counterparts. Following fatigue, significant increases were observed in hip adduction angles, hip adduction moments, and hip flexion moments. Knee abduction angles, knee flexion angles, and knee abduction moments also increased, along with ankle eversion angles, ankle internal rotation angles, and ankle eversion moments. Furthermore, external rotation angles at both the hip and knee joints were notably elevated. During the stance phase of running, the fatigued GV group exhibited greater activation of the quadriceps and gastrocnemius muscles compared to the control group, whereas tibialis anterior (TA) activation decreased. Postfatigue, vastus lateralis (VL) activation further increased, whereas TA activation continued to decline relative to prefatigue levels. These findings underscore the importance of developing targeted exercise interventions to better assess the biomechanical characteristics and potential injury risks associated with GV.

## 1. Introduction

Skeletal deformities of the knee joint are among the most common knee disorders, classified into congenital and acquired types, with genu varum and genu valgum (GV) being the most prevalent [1]. However, some cases of GV or genu varum result from underlying medical conditions such as arthritis, injuries to surrounding knee structures, infections, tumors, tibial growth disorders (Blount's disease), and rickets. These pathological factors can contribute to changes in knee varus and valgus angles [2].

GV, commonly known as “knock-knees,” is an abnormal posture characterized by the knees touching while the ankles remain apart when standing [3]. This condition increases the mechanical load on the lateral compartment of the knee while alleviating stress on the medial compartment [4]. Over time, this imbalance can lead to knee pain, cartilage damage, and early-onset osteoarthritis (OA) [4, 5]. Additionally, it raises the risk of anterior cruciate ligament (ACL) injuries and alters lower limb biomechanics [6, 7]. Research indicates that 70% of ACL tears result from noncontact mechanisms, often involving excessive dynamic valgus, and typically occur when the knee is near full extension [8, 9]. Individuals with GV often exhibit increased knee abduction moments, which impair shock absorption during movement, making them more susceptible to overuse injuries [10].

Additionally, fatigue may impair neuromuscular control, exacerbate load imbalance in individuals with GV [11], increase the risk of knee joint and cruciate ligament injuries, and alter gait kinematics [12, 13]. During drop jumps, individuals with GV exhibit significantly greater changes in knee valgus angles pre and postfatigue compared to healthy individuals [11]. In single-leg landing and running, they demonstrate reduced knee motion in the frontal plane while showing increased hip extension [10, 14]. These pathological factors can lead to alterations in knee varus and valgus alignment [15].

Running is one of the most popular sports [16], and in recent years, marathon and cross-country running have gained significant attention [17, 18]. However, up to 79% of runners experience lower limb injuries related to running [19, 20], most of which are classified as overuse injuries, often attributed to improper training loads [21]. Fatigue during running can lead to gait instability, a reduction in peak vertical ground reaction force (GRF), and a prolonged stance phase, all of which increase the stress on the knee joint [22–25]. Additionally, under fatigue, the hip, knee, and ankle exhibit increased adduction and abduction movements, altering lower limb kinematics [26, 27]. This may result in significant disparities in internal rotation and adduction moments between the knees of healthy individuals.

Previous studies have primarily focused on lower limb biomechanical changes in healthy individuals under fatigue or gait differences in individuals with GV under nonfatigued conditions. As running continues to gain popularity, a deeper understanding of how fatigue affects individuals with GV is crucial for identifying injury risks [28]. Therefore, a significant gap exists in understanding the combined effects of a common stressor like running-induced fatigue on the already altered biomechanics of individuals with GV. Given that running is a highly popular activity closely associated with injuries [29], using a running-induced fatigue protocol is an ecologically valid method to investigate potential injury mechanisms in this at-risk population. Women are particularly prone to GV [30]. Therefore, this study aims to systematically compare changes in lower limb joint angles and joint moments during the stance phase of running between fatigued female individuals with GV and a healthy control group, highlighting biomechanical differences between the two groups. We hypothesize that following running-induced fatigue, females with GV will demonstrate distinct time-series variations in lower limb joint angles and moments during the stance phase, as compared to their healthy counterparts.

## 2. Materials and Methods

### 2.1. Design

**Figure 1:**
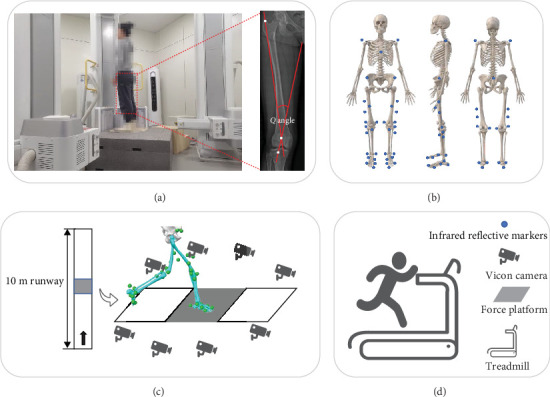
Illustration of the entire experiment. (A) Knee fluoroscopic image data collection and processing using the DFIS. (B) Reflective marker's location. (C) Acquisition of kinematics and kinetics parameters. (D) Running-induced fatigue protocol.

In the present study, each participant was required to complete all experiments within a single day. The experiment consisted of three phases (Figure 1): prefatigue, fatigue protocol, and postfatigue. Kinematic and kinetic data of the lower limbs were collected pre and postfatigue. A running-induced fatigue protocol was implemented between the prefatigue and postfatigue phases, and data collection was performed immediately after completing the fatigue protocol.

### 2.2. Subjects

Before the experiment, G^*⁣*^*∗*^^Power was used to conduct a repeated-measures ANOVA to determine the required sample size (effect size = 0.4, *α* = 0.05, power = 0.8, number of groups = 2, number of measurements = 2, corr among rep measurements = 0.5, nonsphericity correction = 1) [31, 32]. The analysis indicated that 16 participants were needed. As a result, eight female students with GV and eight healthy controls were recruited from Ningbo University. Participants with foot deformities were excluded, and individuals with a history of lower limb injuries or surgeries within the past 6 months were screened out by a physician. All participants were right-leg dominant and had a habit of running as part of their physical activity. Prior to testing, they provided written informed consent. The study protocol received ethical approval from the Human Ethics Committee of Ningbo University (Approval Number: TY2025027).

The GV condition was assessed using the following method: (1) measurement of the quadriceps angle (*Q* angle). A high-speed dual fluoroscopic imaging system (DFIS) was used to capture fluoroscopic images of the participant's knee joint in a static standing position. During data collection, participants maintained an upright posture (Figure 1A), positioning their feet shoulder-width apart while keeping their knees extended and their quadriceps relaxed [33, 34]. The *Q* angle is defined as the angle between two reference lines: one from the patella midpoint to the anterior superior iliac spine, and the other from the patella to the tibial tuberosity. It reflects the direction of the quadriceps' force on the patellar tendon [35]. In healthy females, a normal *Q* angle falls within the range of 15°–20°; exceeding this threshold suggests GV [36]. (2) Intermalleolar distance measurement: participants stood with their lower limbs together, while the gap between the medial malleoli was measured using a calibrated device. A measurement exceeding 0.04 m was considered indicative of GV [37, 38]. The results of these assessments are summarized in Table 1.

### 2.3. Experimental Procedures

The experiment was carried out in a 10-m-long gait laboratory, utilizing a nine-camera infrared Vicon motion capture system (Oxford Metric Ltd., Oxford, UK) and a concave Kistler force platform (Model 9281B, Winterthur, Switzerland) to collect running data. Each participant completed five trials. To reduce measurement errors, all participants wore standardized athletic shoes provided for the study.

Before the experiment, participants' height and weight were recorded using a stadiometer (Seca 213, Hamburg, Germany) and a calibrated weighing scale (Tanita BC-545N, Tokyo, Japan). The study followed a three-phase protocol: prefatigue, fatigue, and postfatigue. Lower limb kinematic and kinetic data were recorded simultaneously during the prefatigue and postfatigue phases. Data collection was conducted using electromyography (EMG) sensors (Trigno; Delsys Inc., Boston, MA, USA), a nine-camera infrared Vicon motion capture system, and a Kistler force platform, with sampling frequencies set at 2000, 200, and 2000 Hz, respectively. EMG sensors were placed on the tibialis anterior (TA), quadriceps femoris, including the vastus lateralis (VL) and vastus medialis (VM), and gastrocnemius comprising the lateral (GL) and medial (GM) heads of the tested limb to measure muscle activity. Electrode placement followed the SENIAM EMG guidelines [39].

Participants wore form-fitting clothing, and 38 reflective markers were placed on their bony landmarks based on the OpenSim 2392 model (Figure 1B) [40]. To maintain consistency, all markers were applied by the same experienced researcher. During both the prefatigue and postfatigue phases, participants ran at a self-selected comfortable pace [41]. Upon the researcher's signal, they ran from the starting point to the finish line. A trial was considered valid if the participant's right foot fully contacted the force platform. To ensure a natural running pattern, researchers assisted in adjusting step placement when necessary.

### 2.4. Running-Induced Fatigue Protocol

Participants first had their lower limb kinematic and kinetic data collected under prefatigue conditions. They then started the fatigue-induction protocol. Perceived exertion was assessed using the 15-point Borg scale [42], and heart rate was tracked with a heart rate sensor (Polar H10; Polar Electro Oy, Kempele, Finland). At the beginning of the protocol, participants started running at an initial speed of 1.1 m/s without additional incline and adapted for 3 min. The speed then increased by 0.45 m/s every minute, reaching 3.3 m/s after 5 min, and was maintained until fatigue. Maximum heart rate was estimated using the formula 220 minus age [43]. Fatigue was determined based on the following criteria: (1) subjective fatigue and experiencing increased respiratory effort, (2) an RPE score of 17, and (3) a heart rate reaching 90% of the estimated maximum. Exhaustion was confirmed if participants continued running for 2 additional minutes after meeting the fatigue criteria.

### 2.5. Data Processing

Marker trajectories were analyzed and processed using Visual 3D software (version 3.26, C-Motion Inc., Germantown, MD, USA). Initially, a static standing trial was utilized to create a subject-specific biomechanical model and to define the neutral (0°) position for each lower limb joint. All joint angles during the dynamic trials were subsequently calculated relative to this static calibration. To eliminate noise, marker trajectories were smoothed using a 4th-order zero-lag Butterworth low-pass filter with a 10 Hz cutoff frequency. Joint angles were then calculated in Visual 3D via inverse kinematics, based on the OpenSim 2392 model, across three planes (sagittal, frontal, transverse). The specific definitions for joint angles were as follows: (1) hip and knee are analyzed in terms of flexion/extension in the sagittal plane (extension defined as positive), adduction/abduction in the frontal plane (abduction defined as positive), and internal/external rotation in the transverse plane (internal rotation defined as positive). (2) Ankle is analyzed in terms of plantarflexion/dorsiflexion in the sagittal plane (dorsiflexion defined as positive), inversion/eversion in the frontal plane (inversion defined as positive), and internal/external rotation in the transverse plane (internal rotation defined as positive). Internal joint moments were derived using inverse dynamics, integrating the kinematic data with synchronized GRF signals, and normalized to body weight (N·m/kg). The stance phase was defined when vertical GRF exceeded 10 N [44], and all data were time-normalized to 101 frames to enable comparison across participants. In this study, the sagittal plane angle of the ankle joint was defined as 0° when the ankle was in full plantarflexion. This definition was based on the coordinate system of the lower leg, using its anatomical structure and movement direction as reference points to determine ankle position and angular changes.

EMGworks Analysis software (Delsys Inc., Boston, MA, USA) processed muscle activation signals by removing the mean value, applying a 10–400 Hz band-pass filter for noise reduction, performing full-wave rectification, and using a 5 Hz low-pass filter. Then, the peak amplitude of the EMG signals recorded during the support phase of running was used to normalize the data [45].

### 2.6. Statistical Analysis

All statistical analyses were conducted using either SPSS (version 27, SPSS, Chicago, IL, USA) or MATLAB (R2024b, The MathWorks Inc., Natick, MA, USA), with the alpha level for statistical significance set at *p* < 0.05. First, the Shapiro–Wilk test assessed the normality of all dependent variables. For discrete variables that followed a normal distribution, parametric tests were applied: independent-samples *t*-tests were used to compare between-group differences between the knee valgus and healthy groups at both the prefatigue and postfatigue time points. A paired-samples *t*-test was used to examine the within-group differences for the knee valgus group from pre to postfatigue. For variables that were not normally distributed, the corresponding nonparametric tests were used: the Mann–Whitney *U* test for between-group comparisons and the Wilcoxon signed-rank test for within-group comparisons. One-dimensional statistical parametric mapping (SPM1D) was utilized for continuous time-series data. This analysis was performed in MATLAB using the SPM1D package (version m.0.4.10, www.spm1d.org) to identify significant differences across the entire movement cycle [46].

## 3. Result

All statistical results are reported below.

### 3.1. Kinematics

**Figure 2:**
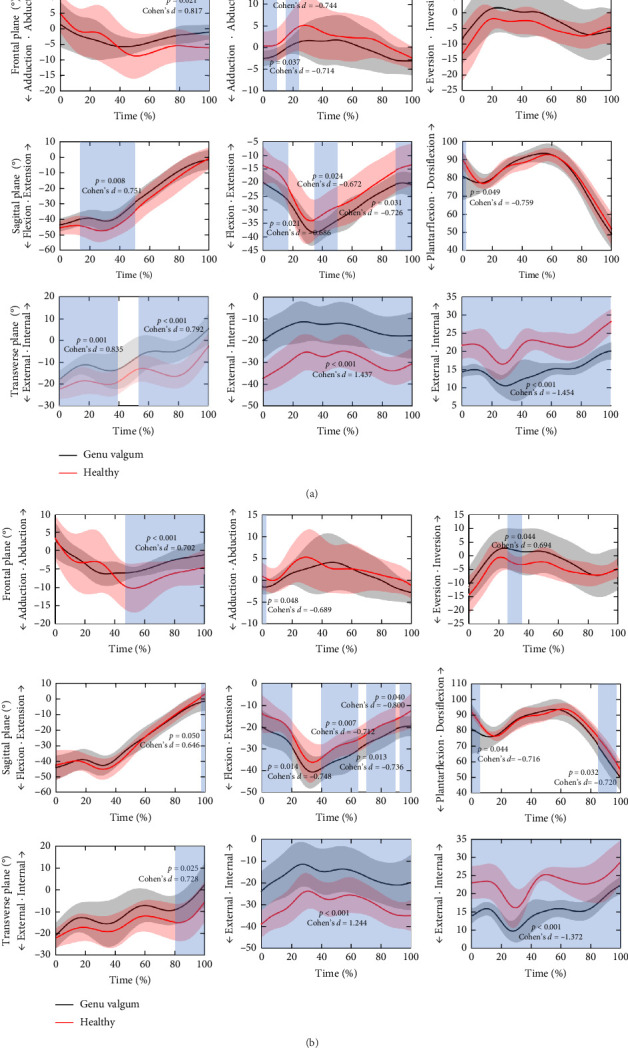
Detailed results of lower limb joint angles in three planes between the GV group and the healthy group. (A) Results of joint angles in the GV group and the healthy group prefatigue. (B) Results of joint angles in the GV group and the healthy group postfatigue. The reported effect size (Cohen's *d*) represents the mean value within the statistically significant interval.

As shown in Figure 2A, prefatigue, the GV group exhibited significantly greater hip abduction angles (77%–100%, *p*=0.021, Cohen's *d* = 0.817), flexion angles (15%–50%, *p*=0.008, Cohen's *d* = 0.751), and internal rotation angles (0%–37%, *p*=0.001, Cohen's *d* = 0.835; 53%–100%, *p* < 0.001, Cohen's *d* = 0.792) compared to the control group. At the knee joint, the GV group demonstrated significantly greater adduction angles (0%–9%, *p*=0.037, Cohen's *d* = −0.714; 18%–23%, *p*=0.039, Cohen's *d* = −0.744) and flexion angles (0%–16%, *p*=0.021, Cohen's *d* = −0.686; 38%–48%, *p*=0.024, Cohen's *d* = −0.672; 92%–100%, *p*=0.031, Cohen's d = −0.726) throughout the stance phase of running. Additionally, their internal rotation remained consistently higher across the entire stance phase (*p* < 0.001, Cohen's *d* = 1.437). For the ankle joint, the GV group showed a greater plantarflexion angle in the sagittal plane between 0% and 4% (*p* < 0.049, Cohen's *d* = −0.759), and consistently larger external rotation during the stance phase compared to the control group (*p* < 0.001, Cohen's *d* = −1.454).

The joint angles of both groups postfatigue are shown in Figure 2B. In the hip joint, compared to the control group, the GV group exhibited greater abduction angles in the frontal plane from 47% to 100% of the stance phase, increased flexion angles in the sagittal plane from 97% to 100%, and significantly higher internal rotation angles in the transverse plane from 81% to 100%. In the knee joint, adduction angles in the frontal plane were significantly higher in the GV group during the initial 0%–4% of the stance phase. Flexion angles in the sagittal plane increased from 2% to 21%, 40% to 65%, 72% to 88%, and 93% to 100%. Additionally, internal rotation angles in the transverse plane remained consistently higher throughout the stance phase. In the ankle joint, the GV group exhibited significantly greater inversion angles in the frontal plane from 26% to 37% (*p*=0.044, Cohen's *d* = 0.694), increased plantarflexion angles in the sagittal plane from 0% to 5% and 87% to 96% (*p*=0.044, Cohen's *d* = −0.716; *p*=0.032, Cohen's *d* = −0.720), and the transverse plane exhibited consistently greater internal rotation angles throughout the stance phase.

**Figure 3:**
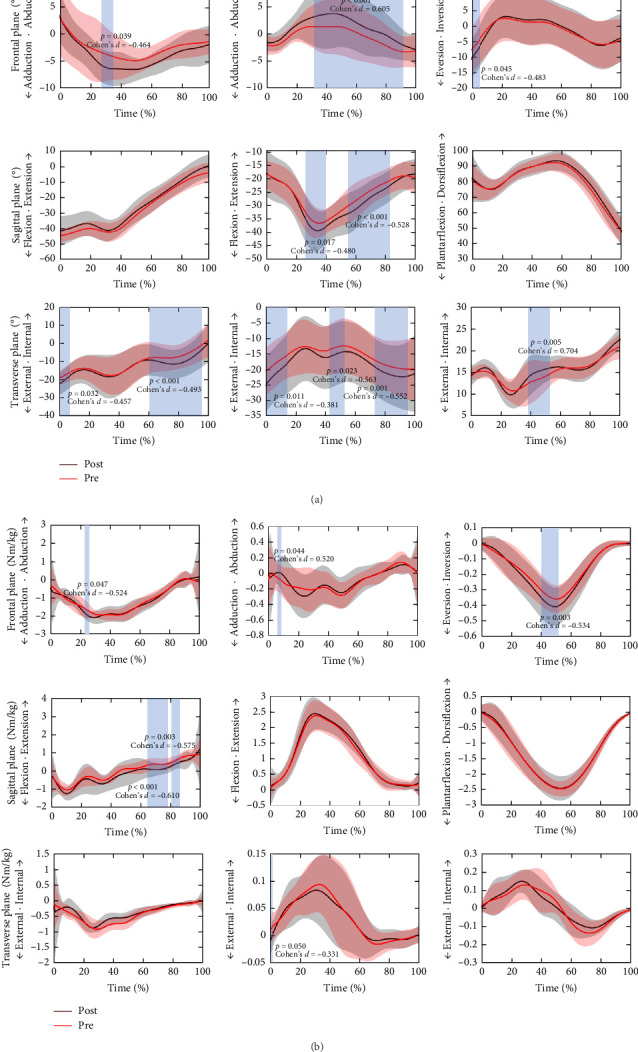
Detailed results of lower limb joint angles and joint moments in three planes of the GV group. (A) Results of joint angles in the GV group pre and postfatigue. (B) Results of joint moments in the GV group pre and postfatigue. The reported effect size (Cohen's *d*) represents the mean value within the statistically significant interval.

Figure 3A presents the variations in lower limb joint angles during the stance phase of running in the GV group pre and postfatigue. Following fatigue, the hip joint demonstrated a significant increase in adduction angles (28%–35%, *p*=0.039, Cohen's *d* = −0.464) and external rotation angles (0%–6%, *p*=0.032, Cohen's *d* = −0.457; 61%–97%, *p* < 0.001, Cohen's *d* = −0.493) compared to the prefatigue state. Likewise, postfatigue, the abduction angles of the knee joint in the frontal plane increased significantly (33%–93%, *p* < 0.001, Cohen's *d* = 0.605), flexion angles in the sagittal plane became more pronounced (28%–39%, *p*=0.017, Cohen's d = −0.480; 55%–83%, *p* < 0.001, Cohen's *d* = −0.528), and external rotation angles in the transverse plane also rose (0%–14%, *p*=0.011, Cohen's *d* = −0.381; 43%–52%, *p*=0.023, Cohen's *d* = −0.563; 73%–95%, *p*=0.001, Cohen's *d* = −0.552). Moreover, the ankle joint exhibited significant alterations in both the frontal and transverse planes. Under fatigued conditions, eversion angles in the frontal plane increased (0%–6%, *p*=0.045, Cohen's *d* = −0.483), along with a rise in internal rotation angles in the transverse plane (39%–53%, *p*=0.005, Cohen's *d* = 0.704) compared to prefatigue measurements.

### 3.2. Kinetics

**Figure 4:**
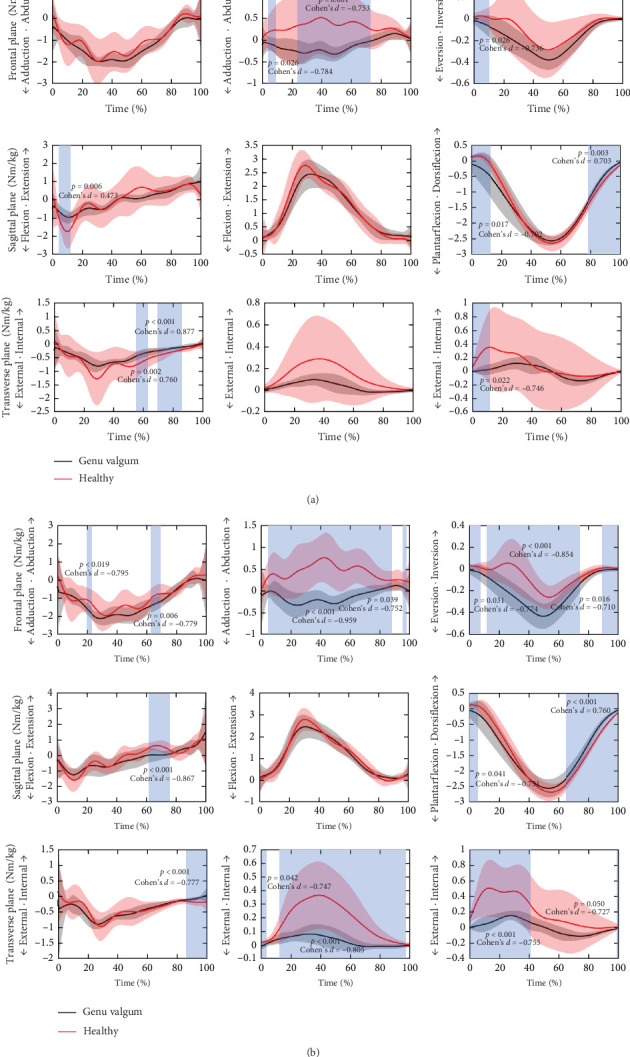
Detailed results of lower limb joint moments in three planes between the GV group and the healthy group. (A) Results of joint moments in the GV group and the healthy group prefatigue. (B) Results of joint moments in the GV group and healthy group postfatigue. The reported effect size (Cohen's *d*) represents the mean value within the statistically significant interval.

During the stance phase of running, significant differences in lower limb joint moments were observed between the GV group and the healthy group prefatigue, as shown in Figure 4A. In the GV group, hip extension moments in the sagittal plane increased (6%–13%, *p*=0.006, Cohen's *d* = 0.473), while external rotation moments in the transverse plane decreased (57%–63%, *p*=0.002, Cohen's *d* = 0.760; 70%–86%, *p* < 0.001, Cohen's *d* = 0.877). The knee joint exhibited significantly greater adduction moments in the frontal plane (5%–10%, *p*=0.026, Cohen's *d* = −0.784; 24%–73%, *p* < 0.001, Cohen's *d* = −0.753). Significant differences between the two groups were observed in the ankle joint across all planes. Prefatigue, compared to the control group, the GV group had relatively smaller inversion moments in the frontal plane (0%–11%, *p*=0.026, Cohen's *d* = −0.736). In the sagittal plane, ankle dorsiflexion moments decreased (0%–13%, *p*=0.017, Cohen's *d* = −0.702) but increased during 79%–100% of the stance phase. Additionally, external rotation moments in the transverse plane significantly increased (0%–12%, *p*=0.022, Cohen's *d* = −0.746).

The lower limb joint moments of the GV and healthy groups postfatigue are shown in Figure 4B. The GV group demonstrated markedly higher hip adduction moments in the frontal plane compared to the healthy group (20%–23%, *p*=0.019, Cohen's *d* = −0.795; 64%–69%, *p*=0.006, Cohen's *d* = −0.779), flexion moments in the sagittal plane (62%–75%, *p* < 0.001, Cohen's *d* = −0.867), and internal rotation moments in the transverse plane (88%–100%, *p* < 0.001, Cohen's *d* = −0.777). The knee joint in the GV group also exhibited significant differences, with reduced abduction moments in the frontal plane (5%–87%, *p* < 0.001, Cohen's *d* = −0.959; 95%–97%, *p*=0.039, Cohen's *d* = −0.752) and increased external rotation moments in the transverse plane (0%–4%, *p*=0.042, Cohen's d = −0.747; 12%–97%, *p* < 0.001, Cohen's *d* = −0.805). Under fatigue, the GV group exhibited increased ankle plantarflexion moments in the sagittal plane (0%–6%, *p*=0.041, Cohen's *d* = −0.731) and significantly greater dorsiflexion moments during 66%–100% of the stance phase. Additionally, the GV group demonstrated significantly greater eversion moments in the frontal plane (0%–8%, *p*=0.031, Cohen's *d* = −0.774; 13%–74%, *p* < 0.001, Cohen's *d* = −0.854; 91%–100%, *p*=0.016, Cohen's *d* = −0.710) and increased external rotation moments in the transverse plane compared to the healthy group (0%–41%, *p* < 0.001, Cohen's *d* = −0.755; 99%–100%, *p*=0.050, Cohen's *d* = −0.727).

As shown in Figure 3B, there are differences in lower limb joint moments in the GV group pre and postfatigue. Following fatigue, the hip abduction moments in the frontal plane were significantly lower than prefatigue (23%–25%, *p*=0.047, Cohen's *d* = −0.524). Conversely, the hip flexion moments in the sagittal plane were significantly higher than prefatigue levels (65%–78%, *p* < 0.001, Cohen's *d* = −0.610; 81%–86%, *p*=0.003, Cohen's *d* = −0.575). Significant changes were also observed in the knee joint. Post-fatigue, the knee abduction moments in the frontal plane (7%–9%, *p*=0.044, Cohen's *d* = 0.520) and the external rotation moments in the transverse plane (1%–3%, *p*=0.050, Cohen's *d* = −0.331) were both significantly greater than prefatigue values. In the ankle joint, fatigue led to a significant increase in the eversion moments in the frontal plane (40%–51%, *p*=0.003, Cohen's *d* = −0.534) in the GV group.

### 3.3. EMG Signals

**Figure 5:**
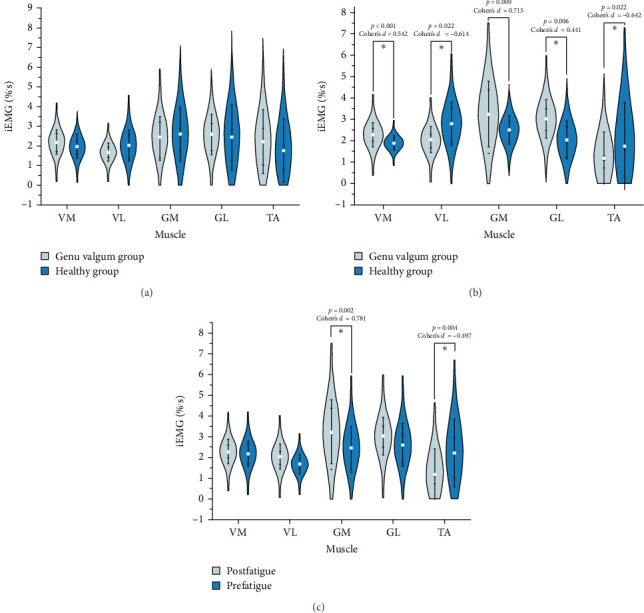
Detailed results of the normalized iEMG parameters of lower limb muscles during the stance phase of running. (A) Results of statistical differences in iEMG parameters during the stance phase of running prefatigue between the healthy and the GV groups. (B) Results of statistical differences in iEMG parameters postfatigue between the healthy and GV groups. (C) Results of statistical differences in iEMG parameters pre and postfatigue in the GV group. All iEMG values were normalized to the peak EMG amplitude during the stance phase and are expressed in percentage per second (%·s). (*⁣*^*∗*^) indicates significant (*p* < 0.05) changes.

As shown in Figure 5, substantial differences were identified between the GV and healthy control groups. Under fatigued conditions, the iEMG activity of the VM (*p* < 0.001, Cohen's *d* = 0.542), VL (*p*=0.022, Cohen's *d* = −0.614), GM (*p*=0.009, Cohen's *d* = 0.715), GL (*p*=0.006, Cohen's *d* = 0.441), and TA (*p*=0.022, Cohen's *d* = −0.642) displayed significant variation between the two groups. Additionally, within the GV group, iEMG activity changed notably pre and postfatigue. In particular, postfatigue GM (*p*=0.002, Cohen's *d* = 0.781) and TA (*p*=0.004, Cohen's *d* = −0.497) activity demonstrated significant differences compared to prefatigue levels.

## 4. Discussion

This study examines the impact of fatigued running on lower limb kinematics and kinetics in females with GV compared to healthy individuals. Following running-induced fatigue, the time-series patterns of joint angles and moments in the lower limbs of individuals with GV deviate from those observed in the healthy control group during the stance phase. These changes may elevate the risk of lower limb injuries, particularly at the knee joint.

The findings of this study support the hypothesis that fatigue induces significant changes in lower limb joint angles and joint moments during the stance phase of running in the GV group. After fatigue, hip adduction (*p*=0.039, Cohen's *d* = −0.464), knee abduction (*p* < 0.001, Cohen's *d* = 0.605), and ankle eversion angles (*p*=0.045, Cohen's *d* = −0.483) increased noticeably compared to prefatigue conditions, likely due to altered muscle activation patterns in the lower limbs. Specifically, gluteus medius activation increased, whereas TA activation decreased, a trend consistent with the findings of Fu et al. [47]. These changes in muscle activation may contribute to a valgus inclination at the ankle and knee joints [48], which, in turn, could disrupt hip alignment by further increasing hip adduction. As a result, gait stability and joint coordination may be compromised. Additionally, reduced neuromuscular control following fatigue may aggravate joint misalignment and elevate the risk of ACL injury [49, 50].

This study observed significant decreases in knee extension angles in individuals with GV before and after fatigue compared to the healthy group (Figure 2). These reductions may be influenced by fatigue and a movement pattern predominantly controlled by noncontractile tissues surrounding the knee joint [51, 52]. As a result, knee extension angles further declined after fatigue, which may be a potential compensatory strategy to absorb impact forces [53]. Additionally, after fatigue, the GV group exhibited increases in ankle plantarflexion moments during the early stance phase of running, followed by declines in the late stance phase. These patterns may be associated with TA fatigue, as reduced TA activation compromised ankle support, disrupting the stability of the total lower limb joint support moments [54, 55]. Furthermore, the study found that the GV group had significantly higher activation of the quadriceps and gastrocnemius muscles after fatigue compared to the healthy group. This suggests that they rely more on these muscles to maintain knee joint stability under fatigued conditions [56]. Since the gastrocnemius and quadriceps play a crucial role in knee flexion and extension [57, 58], their increased activation may provide additional protection to the cruciate ligaments [59, 60] and contribute to overall knee stability [61].

During the stance phase of running, as shown in Figures 2 and 3, those with GV exhibited increased hip flexion angles, abduction angles, and adduction moments after fatigue compared to the healthy group. This may result from a compensatory mechanism triggered by impaired knee joint function. Studies have shown that people with this condition often experience hip abductor weakness [62]. Insufficient strength in these muscles reduces pelvic stability, which may cause excessive knee adduction and lead to an increase in hip flexion angles [63]. In individuals with knee OA, hip abduction moments during gait are typically lower [64], likely due to the influence of passive tension structures such as the iliotibial band [10]. Additionally, those affected by GV often compensate for sagittal plane movement limitations by adjusting movements in the frontal plane [7, 65, 66]. Fatigue may intensify this compensatory strategy, further increasing hip abduction angles [67].

In this study, the GV group exhibited greater knee adduction angles and moments than the healthy group. Research has shown that increased knee adduction angles is associated with OA [68], and people with OA typically experience higher knee adduction moments than those without the condition [69]. Although none of the participants in this study had OA [69], the results suggest that people with GV are still at risk of developing OA. It is important to emphasize that while our findings align with known risk markers, this study was not designed to establish specific clinical thresholds that predict an increased risk of OA. Additionally, compared to the control group, those with GV showed higher ankle plantarflexion moments at heel strike but lower plantarflexion moments at the end of the stance phase. This change may result from altered lower limb alignment due to increased knee adduction angles [70]. Lyle et al. [71] found that expanding the range of motion in ankle plantarflexion and dorsiflexion improves shock absorption at the ankle, thereby reducing the GRF transferred to the knee joint and ACL. Therefore, enhanced ankle mobility in individuals with this alignment issue may help mitigate the negative impact of excessive knee adduction angles and potentially lower their future risk of OA.

Beyond these frontal-plane alterations, it is also necessary to consider the sagittal-plane mechanics, particularly knee flexion, to better interpret the clinical relevance of the observed biomechanical differences. In the GV group, peak knee flexion during stance reached approximately 35°–45°, which lies close to the lower limit of values reported in running biomechanics. Previous studies have reported that the peak knee flexion angle during stance in healthy runners typically falls within the range of 38°–50° [72]. Although the flexion angle in the GV group remained within a typical range, its coexistence with significantly elevated knee adduction angles and moments suggests clinical significance. Elevated adduction loading has been recognized as a key predictor of medial compartment degeneration, with prospective studies showing that peak knee adduction moments exceeding approximately 3.4%BW·Ht are strongly associated with faster progression of medial knee OA [73, 74]. As illustrated in Figure 4, the GV group exhibited significantly greater knee adduction moments compared to the healthy group, underscoring the clinical importance of this biomechanical difference.

Individuals with GV demonstrated larger knee internal rotation angles and higher external rotation moments than the control group. They also exhibited increased hip internal rotation angles and moments. Following fatigue, their knee external rotation angles and moments further increased compared to prefatigue levels (Figure 4). These biomechanical alterations may elevate the risk of ACL injury. Research indicates that female runners with a history of iliotibial band syndrome tend to show reduced knee internal rotation angles [75]. Additionally, movement patterns involving hip internal rotation and knee rotation are closely linked to ACL injury risk [76–80]. Notably, the combination of excessive knee internal rotation and abduction significantly increases ACL strain [81], further heightening the likelihood of injury.

This study provides valuable insights into the lower limb biomechanics of individuals with GV under fatigued conditions. However, certain limitations should be acknowledged. First, this study focused solely on the dominant leg of female participants. The biomechanical characteristics of males with GV may differ from those of females, and both legs contribute to movement during dynamic activities. Therefore, the generalizability of these findings is limited, and caution should be exercised when applying them to other populations or contexts. Second, this study focused only on the stance phase of running, as it is closely associated with running-related injuries [82]. However, individuals with GV may also exhibit significant biomechanical changes during the swing phase, warranting further investigation in future studies. Third, this study only investigated within-group changes before and after fatigue in the GV group. Fatigue-induced changes in the control group were not analyzed, as the main focus was on the fatigue-related biomechanical risks in individuals with GV. Future studies may include within-group analysis of the control group to provide a more comprehensive comparison. Fourth, this study did not evaluate the effectiveness of interventions for individuals with GV. Future research should investigate the protective effects of noninvasive interventions (e.g., Kinesio taping and medial wedge insoles) in managing GV, and utilize finite element analysis to evaluate their biomechanical impact on the musculoskeletal system, such as ACL stress and strain [83–86]. Furthermore, while we report significant kinematic changes, this study did not determine the specific clinical meaningfulness or thresholds for these changes. Future longitudinal studies should aim to establish quantitative relationships between these biomechanical parameters and clinical outcomes. Finally, the EMG data exhibited relatively high interindividual variability, which is a known limitation of surface EMG. Despite applying normalization procedures, differences in neuromuscular activation strategies and electrode placement may still influence signal consistency. Future studies are encouraged to increase sample size and employ high-density EMG systems to improve the accuracy and reliability of muscle activation measurements.

## 5. Conclusions

This study elucidates the effects of fatigue on lower limb biomechanics in female runners with GV compared to a healthy control group. Kinematically, the GV group presented with a pre-existing vulnerability, exhibiting greater hip flexion and internal rotation, alongside increased knee adduction and internal rotation angles even at baseline. Fatigue markedly exacerbated these patterns, inducing a substantial increase in knee abduction, hip adduction, and ankle eversion angles. This altered kinematic profile manifested kinetically as a joint moment profile in the fatigued state. Compared to the control group, the GV group produced significantly higher hip adduction, flexion, and internal rotation moments, as well as greater knee external rotation and ankle eversion moments. A compensatory muscle activation strategy underpinned these mechanical shifts. Within the GV group, fatigue prompted a significant rise in medial GM activation concurrent with a decline in TA activation, contributing to significantly different muscular recruitment patterns compared to the healthy group. Collectively, these interconnected biomechanical alterations suggest an elevated risk of ACL injury in female runners with GV, particularly under fatigued conditions. A limitation of this study is its exclusive focus on female participants. Therefore, future research should broaden the sample population and further investigate the biomechanical mechanisms underlying injury risk in individuals with GV under fatigue conditions.

## Figures and Tables

**Table 1:** Descriptive characteristics of participants.

Variables	GV group		Healthy group	
	Mean	SD	Mean	SD
Age (year)	23.0	1.4	23.0	3.2
Weight (kg)	59.1	11.1	50.3	3.6
Height (m)	1.64	0.09	1.62	0.04
BMI	21.9	1.9	19.3	1.6
*Q* angle	25.3	0.9	17.0	1.4
Distance between two ankles	4.5	0.3	0.5	0.7

Abbreviations: BMI, body mass index; SD, standard deviation.

## Data Availability

All data relevant to the current study are included in the article; further inquiries can be directed to the corresponding author.
